# Navigating Barriers and Building Pathways: Inclusive Education for Children with Special Needs

**DOI:** 10.12688/f1000research.172211.1

**Published:** 2025-11-04

**Authors:** Dalal Aldhilan, Shahid Rafiq, Ayesha Afzal

**Affiliations:** 1 College of Humanities and Social Sciences, Northern Border University, Arar, Northern Borders Province, Saudi Arabia; 2 Department of Social and Behavioral Sciences, Emerson University Multan, Multan, Punjab, 60000, Pakistan; 3 Department of Education, University of Management and Technology, Lahore, Punjab, 54500, Pakistan

**Keywords:** Inclusive education, Special needs, Institutional support, Perceived barriers, Teacher attitudes

## Abstract

This quantitative study investigates the current landscape of inclusive education for children with special needs in Jeddah, Saudi Arabia, with a focus on identifying perceived barriers, assessing institutional support, and quantifying the attitudes of key stakeholders, including teachers, special educators, school administrators, and parents. Drawing on a sample of 550 participants across three major districts of Jeddah, the research utilized a structured questionnaire incorporating Likert-scale items to capture perceptions of inclusion, obstacles to implementation, and the systemic support available in schools. Descriptive analysis revealed generally positive attitudes toward inclusive education (M = 3.85), though moderate levels of perceived barriers (M = 2.90) and institutional support (M = 3.23) suggest significant implementation challenges remain. ANOVA and t-tests showed no statistically significant differences in attitudes across gender or professional roles, indicating a shared understanding of inclusive values across stakeholder groups. However, regression analysis showed no significant predictors of attitudes in a linear model, whereas path analysis revealed that institutional support had a strong positive effect (β = 0.431, p < 0.0001), and perceived barriers had a significant negative effect (β = -0.270, p < 0.0001). These findings underscore the importance of structural and systemic factors in shaping inclusive education outcomes. The study concludes with recommendations for improving institutional support, enhancing teacher training, and fostering inclusive infrastructure, while also noting limitations related to geography and self-reporting. The research contributes to Saudi Arabia’s broader Vision 2030 goals by offering evidence-based insights for creating a more inclusive and equitable education system.

## Introduction

Inclusive education has increasingly become a global benchmark for equitable, just, and democratic schooling systems. Its premise, that all learners, regardless of their abilities or disabilities, have the right to learn together in regular educational settings, has transformed pedagogical paradigms and institutional frameworks across continents. The movement gained international momentum following the UNESCO Salamanca Statement (1994), which urged nations to prioritize inclusive schooling and to adapt systems to the needs of all learners. Over the last two decades, inclusive education has evolved from a rights-based ideology into a measurable educational goal across global educational policies. The implementation of inclusive practices reflects the maturity of educational systems and their capacity to cater to diversity, particularly the needs of children with disabilities and special learning needs. In this global context, the Kingdom of Saudi Arabia (KSA) has shown commendable ambition and intent. With the launch of Vision 2030, the country aims to modernize its educational infrastructure and diversify learning to ensure that all citizens, regardless of gender, ability, or geography, have equal access to quality education. One of the three central themes of Vision 2030 is “A Thriving Economy,” which places strong emphasis on educational empowerment and inclusive development (Ministry of Education, 2019). Central to this ambition is the notion that children with disabilities and special needs must not be left behind. Accordingly, inclusive education has emerged as a strategic goal within Saudi Arabia’s national development agenda.

However, while the vision is bold, its realization on the ground faces considerable challenges, particularly in urban centers like Jeddah, which are home to diverse populations and rapidly expanding school systems. The infrastructure, teacher readiness, societal attitudes, and pedagogical practices do not yet uniformly support inclusive learning. Research has shown that, despite inclusive policies, children with disabilities in Saudi Arabia often encounter exclusion from mainstream education due to attitudinal, institutional, and logistical barriers ( Alsolami, 2024). For example, a lack of specialized training among teachers, inaccessible classroom environments, and negative peer perceptions are frequently cited issues ( Alsolami *,* 2024). These issues are not unique to Saudi Arabia. Globally, the transition from segregated special education to inclusive classrooms is fraught with systemic resistance, resource limitations, and professional capacity gaps. However, the local socio-cultural and policy contexts in Saudi Arabia amplify these challenges. In Jeddah, a city with considerable economic activity and educational diversity, inclusion is theoretically attainable but practically uneven. Many schools lack clear implementation frameworks, inclusive curricula, or monitoring and evaluation tools to ensure effective integration of special needs students ( Medabesh et al., 2024).

### Problem statement

The problem thus lies not in the absence of vision or intent, but in the persistent barriers that obstruct inclusive practices at the classroom, school, and policy levels. These include, but are not limited to, negative attitudes of teachers and school staff, inadequate training, insufficient assistive technology, and a lack of collaboration between schools and families. A recent review by Al-Shehri (2021) emphasized that despite increasing awareness, teachers in cities like Jeddah still report low confidence and preparedness in handling inclusive classrooms. Furthermore, logistical and architectural obstacles, such as a lack of ramps, elevators, or adapted instructional materials, contribute to a continued marginalization of students with physical and cognitive disabilities in regular schools.

Given these challenges, there is an urgent need to examine how inclusive education is being practiced and perceived in Jeddah. This research study is therefore designed to explore and quantify the attitudes, experiences, and perceived barriers among key stakeholders: teachers, school administrators, and families of children with special needs. A quantitative approach is essential to generate statistically significant findings that can inform policy adjustments and future training programs.

### Research objectives

The primary objectives of this study are twofold. First, it seeks to identify and analyze the key barriers to implementing inclusive education in public and private schools in Jeddah. This includes both structural and attitudinal obstacles. Second, it aims to quantify the attitudes of educators and parents toward inclusive practices, whether they perceive inclusion as beneficial or burdensome, and under what conditions it can be effectively realized.

### Research questions

To achieve these objectives, the following research questions guide this inquiry:

1. What are the key barriers to inclusive education in Jeddah as perceived by educators and families?
2. This question seeks to categorize and prioritize the most prevalent challenges, ranging from training deficits and physical accessibility to curriculum flexibility and administrative support. Existing studies have identified common issues, but localized data from Jeddah is limited and urgently needed ( Abed, 2020).
3. How do teachers and school administrators perceive the inclusion of children with special needs in mainstream classrooms?
4. This question aims to uncover the prevailing beliefs, biases, and attitudes that influence teachers’ willingness and preparedness to accommodate diverse learners. For instance, Alsamiri et al. (2022) noted that while many teachers agreed with the concept of inclusion, they felt ill-equipped to practice it effectively due to a lack of training and support ( Alsamiri et al., 2022).
5. What infrastructural, social, or pedagogical pathways currently exist, or are lacking, that influence the success of inclusive education in Jeddah?
6. By addressing this question, the study assesses the systemic supports (e.g., teacher professional development, accessible learning environments, parent engagement strategies) and identifies gaps that must be bridged to promote successful inclusion.

This study not only builds upon existing national and international literature but also attempts to fill a significant gap in localized, empirical knowledge about inclusion in Jeddah. Its results contribute to a more informed conversation on how best to operationalize inclusive education within Saudi Arabia’s broader educational reforms under Vision 2030. Ultimately, the study aims to empower policymakers, school leaders, and educators with actionable insights so that inclusive education in Jeddah becomes not just an aspiration but a lively reality for all children.

## Literature review

Inclusive education has become a dominant principle in international educational discourse, supported by global frameworks such as the UNESCO Salamanca Statement (1994) and the United Nations’ Sustainable Development Goal 4, which advocates for “inclusive and equitable quality education for all.” These frameworks emphasize that inclusive education should not only accommodate children with disabilities but should also embrace all forms of diversity, including those based on language, ethnicity, socioeconomic status, and learning styles. As inclusion has become institutionalized in global policy agendas, attention has shifted from access to meaningful participation, requiring significant pedagogical and structural reforms in educational systems. Ainscow (2020) emphasizes the importance of promoting equity within schools as a foundation for inclusive education. He introduces a multidimensional model that urges schools to examine their cultures, policies, and practices to reduce barriers to learning and participation. His work underscores the idea that inclusion is not simply a technical issue to be resolved with additional resources, but a moral and political imperative requiring collaborative action within education systems ( Ainscow, M. (2020). Promoting equity in schools. Routledge). Florian and Spratt (2013) also argue for a shift in pedagogical thinking, moving away from viewing inclusion as an add-on and instead promoting “inclusive pedagogy,” where teachers proactively plan for learner variability rather than retrofitting instruction ( Florian & Spratt, 2013).

In high-income nations such as Sweden, Finland, and Canada, inclusive education is characterized by strong policy backing, extensive teacher training, and school-level autonomy. However, even in these settings, challenges persist, particularly in aligning inclusive philosophies with standardized assessment systems and rigid curricula. Global research also points to the continued marginalization of students with multiple disabilities, language barriers, or those from minority ethnic groups, showing that inclusive education must be seen as an ongoing process rather than a static goal. The global landscape provides both inspiration and cautionary tales for countries like Saudi Arabia that are in transitional stages of inclusive reform.

### Inclusive education in the Arab world

In the Arab region, the implementation of inclusive education has gained traction over the last two decades, especially in light of commitments made under international conventions. However, inclusion remains a policy ambition rather than an entrenched educational practice in many countries. Cultural attitudes toward disability, traditional teaching methods, underdeveloped support services, and a lack of specialist training are frequently cited as barriers across the region.

In a landmark study, Alnahdi et al. (2019) examined the evolution of special education in Saudi Arabia, highlighting both legal advancements and practical challenges. He notes that although the Saudi government has enacted legislation to support inclusive education, implementation across schools remains inconsistent due to a lack of coordination between ministries and inadequate funding for necessary resources such as assistive technologies and classroom aides ( Alnahdi et al., 2019).

Teacher preparedness is a major obstacle throughout the Arab world. Many general education teachers report low self-efficacy in dealing with students with disabilities due to insufficient training. Alkhunini (2025) emphasized that in countries like Oman and Kuwait, teachers often lack access to professional development focused on inclusive strategies, which leads to a dependence on special educators and creates a parallel rather than integrated model of education ( Alkhunini, 2025). Furthermore, societal beliefs, shaped by religious, cultural, and familial expectations, can either support or hinder inclusive practices, especially when disability is seen as a source of shame or stigma.

Family involvement is another area of concern. In many Arab countries, there is limited collaboration between schools and families of children with disabilities. Parents often feel excluded from educational planning and decision-making processes. Khalil and Yasmeen (2020) point out that in the MENA region, awareness of inclusive rights and services is still limited among families, and many lack the confidence or knowledge to advocate effectively for their children ( Khalil & Yasmeen, 2020). Despite these challenges, there have been promising reforms in the region. Countries like the UAE and Qatar have made significant strides in enacting inclusive legislation, investing in teacher training, and creating specialized support centers. However, scalability and sustainability remain pressing issues. The Arab world presents a complex mix of progressive policy intentions and deeply entrenched structural and cultural barriers, lessons that are highly relevant for the case of Saudi Arabia and, more specifically, for urban centers like Jeddah.

### Specific case of Saudi Arabia and Jeddah

Saudi Arabia’s inclusive education journey has been significantly influenced by its national development blueprint, Vision 2030, which explicitly mentions enhancing education for all, including individuals with disabilities. The Ministry of Education has initiated several programs to integrate students with special needs into mainstream schools, encouraged the development of resource rooms, and established centers for special education services. However, these efforts have encountered mixed results in terms of execution at the school level.

Jeddah, as one of the Kingdom’s most populous and diverse cities, represents both opportunities and challenges in the realm of inclusion. On one hand, it hosts several modern schools that are well-resourced and innovative. On the other hand, many schools lack the physical infrastructure, trained personnel, and inclusive leadership required for successful implementation. Research by Alsolami (2025) investigated the use of artificial intelligence (AI) in enhancing the academic skills of students with mild intellectual disabilities in Jeddah. The study found positive outcomes in student performance when AI tools were used for personalized learning, particularly in reading and mathematics. However, the study also revealed that such tools were used in only a handful of elite schools, reflecting systemic inequities in access to educational technologies ( Alsolami, 2025). The effectiveness of using artificial intelligence in improving the academic skills of school-aged students with mild intellectual disabilities in Saudi Arabia. Research in Developmental Disabilities. Other studies have echoed similar concerns. Alrawkan (2022) conducted a multi-study review across Saudi public schools and found that although policy documents highlight inclusive goals, there is often a lack of practical guidance and accountability mechanisms at the school level ( Alrawkan, 2022). The situation in Jeddah mirrors this national picture; school leadership plays a decisive role in determining the degree of inclusion, and in many cases, school principals lack the training to lead inclusive transformations.

Teacher attitudes continue to pose a major challenge in Jeddah’s schools. Many teachers view inclusive education as an additional burden due to a lack of support, training, and time. Teachers expressed willingness to support inclusion but cited structural issues like overcrowded classrooms, lack of differentiated materials, and minimal collaboration with specialists as hindrances ( Alqahtani, 2017). Teacher perspectives on full inclusion of students with learning disabilities in Saudi Arabian high schools. Indiana State University. These findings are consistent with Alshehri’s (2023) research in Jeddah, which highlighted that both general and special education teachers experienced frustration when working in isolation rather than as part of a collaborative inclusion team ( Alshehri, 2023). Furthermore, despite increased investment in infrastructure, many schools in Jeddah still lack essential accessibility features such as ramps, elevators, and adapted furniture. Curricula also remain rigid and exam-focused, making it difficult to implement the flexible, differentiated instruction that inclusive education demands. In this context, there is a critical need for systematic reforms that address not only policy but also practice, from leadership and teacher development to parental engagement and curriculum redesign.

### Theoretical framework

**Figure 1.**
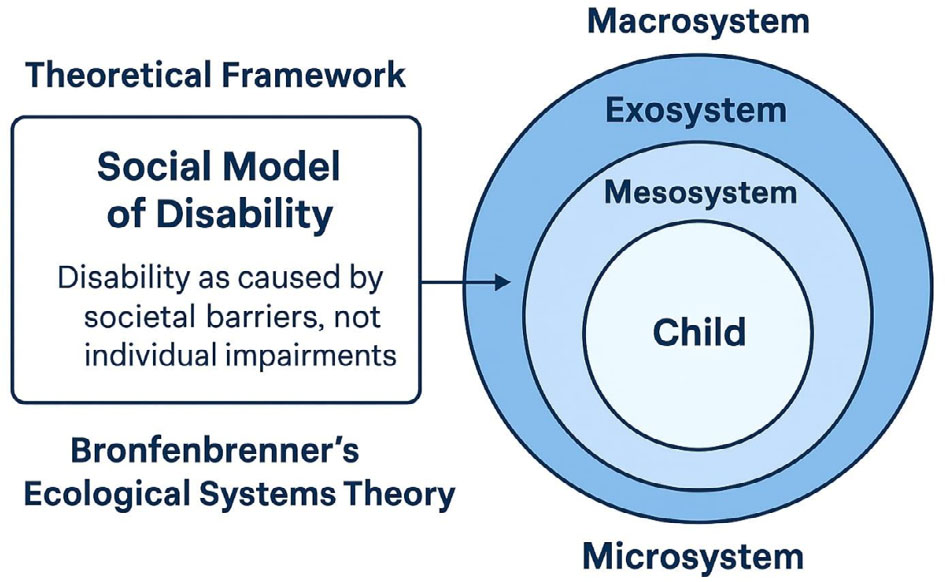
Theoretical framework.

This study is underpinned by two interrelated theoretical frameworks: the Social Model of Disability and Bronfenbrenner’s Ecological Systems Theory. Together, these perspectives provide a comprehensive understanding of the systemic, environmental, and social factors influencing inclusive education for children with special needs in Jeddah, Saudi Arabia, as shown in Figure 1.

The Social Model of Disability asserts that disability is not caused by an individual’s impairments but by societal barriers, physical, attitudinal, and institutional, that restrict participation and inclusion ( Shakespeare, 2013). This model reorients attention away from the child’s condition and toward the responsibility of educational institutions to remove obstacles to learning and participation. Within this framework, exclusion is viewed as a product of inflexible curricula, inaccessible buildings, and insufficient teacher training, rather than a consequence of the child’s disability. This perspective aligns with the aims of Saudi Arabia’s Vision 2030, which emphasizes the creation of inclusive environments that promote equal access to education and societal integration for individuals with disabilities ( Alnahdi et al., 2019). In the context of this study, the Social Model provides a critical lens for identifying how school systems in Jeddah may perpetuate exclusion through their structures, policies, or lack of accommodations. It also informs the need to evaluate the attitudes and readiness of educators to shift toward a more inclusive paradigm.

To complement this, Bronfenbrenner’s Ecological Systems Theory provides a dynamic view of how multiple environmental systems interact to shape a child’s educational experience. Bronfenbrenner (1979) describes development as occurring within nested systems: the microsystem (e.g., child, family, teacher), mesosystem (interactions between school and home), exosystem (school policies, professional development), and macrosystem (cultural values, national policies). In Saudi Arabia, these levels are evident in the interaction between national education reforms, school leadership, family engagement, and teacher beliefs. For instance, a child’s success in inclusive education may depend not only on their teacher’s practices but also on the school’s infrastructure, family involvement, and the Ministry of Education’s policy support. Together, these two frameworks allow for an analysis that is both structural and contextual. They inform the study’s design by emphasizing the importance of assessing barriers across multiple levels, from teacher attitudes and school environments to broader cultural and policy frameworks.

## Methodology and procedure

This study adopts a positivist research paradigm, which is widely used in social science research for its emphasis on objectivity, quantification, and generalizability ( Creswell & Creswell, 2017). The positivist approach aligns with the goals of this study, to systematically quantify and analyze the perceptions, attitudes, and perceived barriers to inclusive education among different stakeholder groups in Jeddah, Saudi Arabia. Positivism is particularly suitable for educational research when the aim is to identify patterns, test relationships, and inform policy through statistically valid conclusions ( Mertens, 2019). By adopting this paradigm, the research remains focused on empirically observable data, free from researcher bias or interpretive subjectivity. Based on this philosophical orientation, the study employs a quantitative descriptive research design. Descriptive research is appropriate when the objective is to describe the characteristics of a population, measure the prevalence of certain phenomena, and identify associations between variables ( Cohen et al., 2018). A survey method is used for data collection, as it allows for gathering standardized data from a large and diverse group of participants. The survey design also facilitates the collection of information on attitudes, perceptions, and reported experiences, making it ideal for investigating the multifaceted issue of inclusive education implementation.

The use of a cross-sectional approach enables the collection of data at a single point in time, which is suitable for capturing current conditions and stakeholder sentiments regarding inclusive education in Jeddah. The findings from this approach help in identifying common barriers and support mechanisms as experienced by school personnel and parents, providing valuable insights for practitioners and policymakers.

### Participants and sampling

The study targets four main stakeholder groups: schoolteachers, special educators, school principals, and parents of children with and without special needs. A total of 550 participants (N = 550) were selected from schools located across the three main regions of Jeddah, North, South, and Central, to ensure geographic and demographic diversity. To achieve a representative sample, the study uses stratified random sampling. Stratification is based on school type (public, private, and international) and geographic location. This method ensures that subgroups are proportionally represented, increasing the generalizability of the findings ( Bryman, 2016). Within each stratum, participants are selected randomly from a database of schools provided by the Ministry of Education in Jeddah. The stratified approach also mitigates sampling bias and improves external validity.

The inclusion criteria for participation include:

• For educators and administrators: Employment in a school located in Jeddah, with at least one year of experience.
• For parents: Having at least one child enrolled in a school in Jeddah.
• Special consideration was given to ensure adequate representation of both male and female participants, as gender roles in education remain influential in Saudi society ( Alnahdi et al., 2019).

### Instrumentation

The primary tool for data collection is a structured, self-administered questionnaire, developed based on previous validated instruments used in similar contexts ( Forlin & Sin, 2010; Alkhunini, 2025). The questionnaire is designed to measure three core constructs:

1. Attitudes toward inclusive education
2. Perceived barriers to inclusion
3. Institutional and policy-level support mechanisms

Each section of the questionnaire uses a 5-point Likert scale, ranging from “Strongly Disagree” (1) to “Strongly Agree” (5). Likert-scale-based items are commonly used in educational research to assess subjective variables, such as attitudes and perceptions, and are conducive to both descriptive and inferential analysis ( Boone & Boone, 2012).

• Attitude items explored participants’ beliefs about the benefits and feasibility of inclusive education (e.g., “Inclusion benefits all students socially and academically”).
• Barrier items measured perceptions of obstacles (e.g., “I feel unprepared to teach students with disabilities” or “There is a lack of resources in my school”).
• Support items assess the extent of institutional backing (e.g., “My school provides training on inclusive education” or “Government policies are clear and supportive”).

To ensure content validity, the draft questionnaire was reviewed by three experts in inclusive education and psychometrics from King Abdulaziz University. Minor revisions were made to align item wording with cultural norms in Saudi Arabia. A pilot study was conducted with 30 participants to test the clarity, reliability, and internal consistency of the instrument. Based on the pilot data, Cronbach’s alpha values for all subscales exceeded 0.80, indicating strong reliability.

### Ethical procedures and data collection

This study was conducted in accordance with the ethical standards outlined in the Declaration of Helsinki and approved by the Deanship of Scientific Research Ethical Committee at Northern Border University, Saudi Arabia (Approval Reference: NBU-DSR/IRB/2024/128). All participants were adults and provided with written informed consent prior to participating in the study. No minors were involved in this research.

Data was collected through both online and paper-based formats to maximize accessibility and response rates. Online surveys were distributed via official school emails and parent groups, while paper versions were distributed to schools in under-resourced areas without stable internet access. Surveys were available in both Arabic and English, ensuring linguistic inclusivity. Participants were assured of their anonymity, and all data were stored in password-protected files accessible only to the research team. The data collection period spanned six weeks, with regular follow-up reminders sent to participating institutions to ensure sufficient response rates across all strata.

### Data analysis

Quantitative data collected through the structured survey were analyzed using SPSS (Statistical Package for the Social Sciences). The analysis began with descriptive statistics to summarize participant demographics and central tendencies of the core study variables. Frequencies and percentages revealed a balanced sample distribution: 56.55% of participants were female, and 43.45% were male. In terms of roles, teachers made up the largest group (43.27%), followed by parents (27.09%), special educators (20.55%), and school principals (9.09%). Geographically, participants were fairly evenly distributed across North (31.82%), South (33.64%), and Central Jeddah (34.55%).

The descriptive analysis showed that attitudes toward inclusive education had a relatively high mean score of 3.85 (SD = 0.80), translating to approximately 76.95% positivity. Perceived barriers had a moderate mean score of 2.90 (SD = 0.82), indicating a notable presence of structural or pedagogical constraints, while institutional support yielded a mean of 3.23 (SD = 0.84) or 64.51%, suggesting room for improvement in support mechanisms.

To examine subgroup differences, inferential statistics were applied. A one-way ANOVA tested whether attitudes differed across professional roles. The results indicated no significant differences across groups (F = 0.25, p = 0.8612), suggesting a broadly consistent perspective on inclusive education irrespective of professional designation. Additionally, a t-test comparing attitudes by gender revealed no statistically significant difference between male (M = 3.79) and female (M = 3.89) participants (t = -1.35, p = 0.1779).

A correlation matrix was computed to assess the relationships between the three key constructs. Weak correlations were observed: attitudes were slightly negatively correlated with institutional support (r = -0.07) and slightly positively correlated with perceived barriers (r = 0.07), while institutional support and barriers were negatively related (r = -0.04). These low correlations prompted further modeling to understand the predictors of inclusive attitudes.

A multiple regression analysis was conducted to determine whether barriers and institutional support significantly predicted attitudes toward inclusive education. The results revealed that neither variable had a statistically significant predictive effect when modeled together: barriers (β = 0.066, p = 0.1097) and institutional support (β = -0.059, p = 0.1435). While directionality was observed, the findings suggested possible multicollinearity or confounding interactions not captured through linear regression.

To further clarify the directional influences and account for standardized relationships, a Path Analysis was performed using standardized variables. This model showed stronger and statistically significant relationships. Perceived barriers negatively predicted attitudes (β = -0.270, p < 0.0001), while institutional support positively predicted attitudes (β = 0.431, p < 0.0001). These results provided robust evidence that, when properly scaled and modeled, the institutional and structural context plays a crucial role in shaping stakeholders’ perceptions of inclusive education.

Overall, the statistical analysis confirmed that while demographic factors such as gender or role do not significantly affect attitudes toward inclusion, perceived institutional support and the presence of barriers exert meaningful influences. The use of Path Analysis clarified these relationships more effectively than traditional regression, offering deeper insights into the systemic factors driving inclusive education in the context of Jeddah.

All statistical analyses were conducted at a 95% confidence level, and effect sizes and p-values were reported to ensure transparency and scientific rigor. These findings were interpreted in light of the existing literature and policy landscape of Saudi Arabia, providing empirical grounding for recommendations and future action.

**Figure 2.**
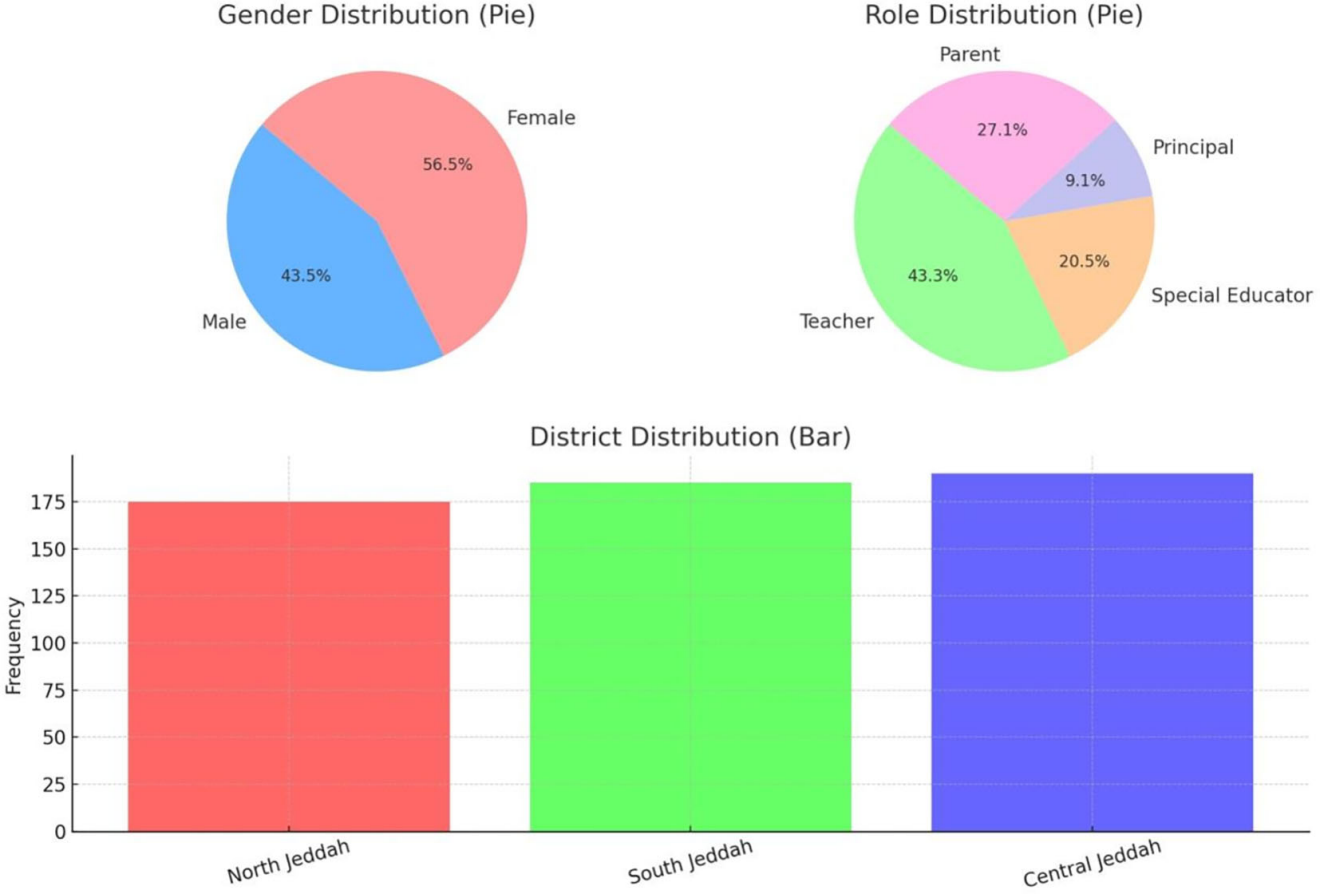
Demographic characteristics of respondents.

The demographic data in Table 1 show a balanced gender distribution, with 56.55% female and 43.45% male participants, indicating diverse gender perspectives in the study. The largest respondent group was teachers (43.27%), followed by parents (27.09%), reflecting strong representation from those directly involved in inclusive education. Additionally, participants were fairly evenly distributed across North, South, and Central Jeddah, enhancing the geographic representativeness of the findings, as shown in Figure 2 below.

**Table 1.** Demographic information of respondents.

Variable	Category	Frequency	Percentage
Gender	Male	239	43.45%
	Female	311	56.55%
Role	Teacher	238	43.27%
	Special Educator	113	20.55%
	Principal	50	9.09%
	Parent	149	27.09%
District	North Jeddah	175	31.82%
	South Jeddah	185	33.64%
	Central Jeddah	190	34.55%

The descriptive statistics in Table 2 indicate that respondents generally hold positive attitudes toward inclusive education, with a high mean score of 3.85 (76.95%). However, the mean score for perceived barriers (2.90) suggests that moderate obstacles still hinder effective implementation. Meanwhile, institutional support scored 3.23 (64.51%), reflecting a moderate level of support, which may not be sufficient to fully facilitate inclusive practices. These findings highlight the need to strengthen institutional frameworks while addressing existing barriers.

**Table 2.** Descriptive statistics.

Variable	Mean	SD	Min - Max	Mean as %
Attitudes	3.85	0.80	1-5	76.95%
Barriers	2.90	0.82	1-5	57.96%
Institutional Support	3.23	0.84	1-5	64.51%

The ANOVA results in Table 3 reveal that there is no statistically significant difference in attitudes toward inclusive education across different respondent roles (teachers, special educators, principals, and parents), as indicated by an F-value of 0.25 and a p-value of 0.8612. This suggests a broad consensus across stakeholder groups, indicating that despite their differing roles, participants generally share similar perceptions about inclusive education.

**Table 3.** ANOVA - Attitudes across roles.

Source	Sum squares	df	F (p-value)
Between Groups	0.48	3	0.25 (p = 0.8612)
Within Groups	348.69	546	-

The independent samples t-test in Table 4 shows no statistically significant difference in attitudes toward inclusive education between male (M = 3.79) and female (M = 3.89) participants, with a t-value of -1.35 and p = 0.1779. Although females reported slightly more positive attitudes, the difference is not meaningful at the 0.05 significance level, suggesting that gender does not significantly influence perceptions of inclusive education in this sample.

**Table 4.** T-test - Gender differences in attitudes.

Group	Mean attitudes	n
Male	3.79	239
Female	3.89	311

T-Statistic = -1.35, p = 0.1779.

The correlation matrix in Table 5 reveals very weak relationships between the three variables. Attitudes are slightly positively correlated with barriers (r = 0.07) and slightly negatively correlated with institutional support (r = -0.07), while barriers and institutional support show a very weak negative correlation (r = -0.04). None of these correlations suggest meaningful linear relationships, indicating that the variables may interact in more complex, non-linear ways, justifying the need for advanced modeling like regression or path analysis.

**Table 5.** Correlation matrix.

Variables	Attitudes	Barriers	Institutional support
Attitudes	1.00		
Barriers	0.07	1.00	
Institutional Support	-0.07	-0.04	1.00

The regression analysis in Table 6 shows that neither perceived barriers nor institutional support significantly predicts attitudes toward inclusive education. The coefficient for barriers (β = 0.066, p = 0.1097) and institutional support (β = -0.059, p = 0.1435) both fall above the conventional 0.05 significance threshold, indicating that these variables do not individually explain meaningful variance in attitudes within a linear model. While the intercept is statistically significant, the predictors’ lack of significance suggests that other factors or more complex interactions may be influencing attitudes, which warrants further analysis through path modeling.

**Table 6.** Regression Analysis - Predicting attitudes.

Predictor	Coef.	Std Err	t	p
Intercept	3.846	0.184	20.87	0.0000
Barriers	0.066	0.041	1.60	0.1097
Institutional Support	-0.059	0.040	-1.46	0.1435

**Figure 3.**
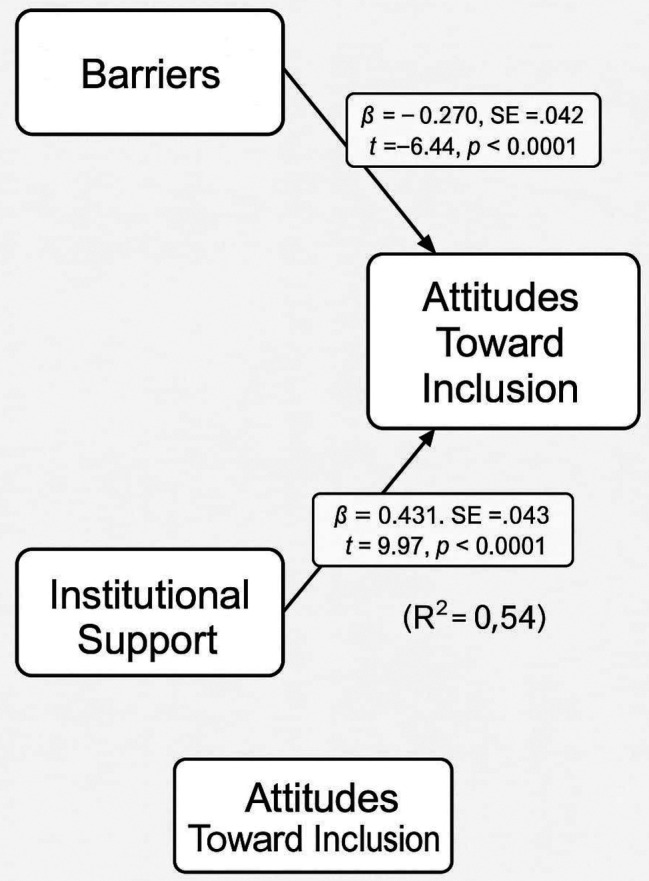
Path analysis model predicting attitude.

The path analysis in Table 7 reveals that both barriers and institutional support are statistically significant predictors of attitudes toward inclusive education when modeled together using standardized coefficients. Specifically, perceived barriers have a significant negative effect on attitudes (β = -0.270, p < 0.0001), suggesting that as barriers increase, positive attitudes toward inclusion decrease. Conversely, institutional support shows a strong positive influence on attitudes (β = 0.431, p < 0.0001), indicating that greater support enhances favorable views of inclusion. The intercept is not significant, as expected in a standardized model. These findings confirm the value of path analysis over simple regression, revealing robust causal pathways between institutional dynamics and stakeholders’ attitudes, as shown in Figure 3.

**Table 7.** Path analysis results.

Path	(β)	SE	t-value	p-value
Barriers → Attitudes	-0.270	0.042	-6.44	<0.0001
Institutional Support → Attitudes	0.431	0.043	9.97	<0.0001
*(Intercept/Constant)*	~0.000 (baseline)	0.040	0.01	0.990

## Discussion

The findings of this study, centered on inclusive education in Jeddah, reveal meaningful insights when situated within global and regional academic discourse. Most notably, the positive attitudes held by a majority of respondents toward inclusive education echo global trends that recognize inclusive education as a fundamental human right and a driver of educational equity ( Ainscow, 2020; Florian & Spratt, 2013). The high mean score for attitudes (M = 3.85) in this study suggests a foundational acceptance of inclusive principles among stakeholders, teachers, special educators, principals, and parents. This aligns with research in other contexts that emphasize the growing consensus around inclusive education, such as in the United Kingdom, Australia, and parts of Southeast Asia ( Forlin et al., 2013).

However, a key divergence from some international literature emerges in the regression and correlation results, where neither barriers nor institutional support significantly predicted attitudes in the initial regression model. This may be due to multicollinearity or complex underlying mechanisms not captured in linear analysis. Interestingly, the path analysis clarified these relationships, revealing that institutional support positively influences attitudes (β = 0.431), while perceived barriers have a significant negative effect (β = -0.270). These findings resonate with studies from the Gulf and MENA regions, where systemic support and resourcing are often seen as more critical than individual effort in fostering inclusive schooling ( Almalky & Alwahbi, 2023). The weak correlations observed among variables, particularly between attitudes and institutional support, may reflect the complexity of inclusion in the Saudi context. Unlike Western education systems, which often have long-established inclusive policies, Saudi Arabia is still in the process of transitioning toward inclusive models under the broader umbrella of Vision 2030. Vision prioritizes educational reform and acknowledges the need for inclusivity, but local implementation, particularly in urban districts like Jeddah, faces infrastructural, pedagogical, and societal challenges ( Ahmed, 2021).

This study also aligns with findings from Almalky and Alwahbi (2023), who note that while inclusive education is promoted at the policy level in Saudi Arabia, school-level execution is hindered by limited teacher training and unclear curricular adaptations. Additionally, cultural norms and stigma around disability continue to shape perceptions, a factor reported in both regional and global studies ( Crabtree, 2006). Thus, while the attitudinal climate is positive, it remains vulnerable to institutional inconsistencies and cultural inertia.

### Implications

The results of this study have several significant implications for education policy, school practice, and teacher development within Jeddah and, more broadly, Saudi Arabia. First and foremost, the finding that institutional support strongly predicts inclusive attitudes underlines the urgent need for systemic reform. Schools cannot achieve inclusiveness through isolated efforts; rather, they require coordinated support in terms of leadership, funding, infrastructure, and legal enforcement. Ministries and school districts should allocate dedicated resources toward inclusive policies, such as the provision of assistive technologies, hiring of qualified special educators, and retrofitting of physical environments for accessibility ( Ahmed, 2021).

From a policy perspective, these findings advocate for a national inclusion strategy that goes beyond rhetoric to include measurable indicators of progress and compliance. For example, educational authorities can adopt performance frameworks similar to those used in countries like Finland or Canada, where schools are evaluated based on inclusion benchmarks. Policy must also include clear mandates for data collection on students with disabilities, a current gap that hinders evidence-based planning in Saudi Arabia ( Alzahrani, 2021). In terms of teacher development, the study’s findings clearly support calls for enhanced pre-service and in-service training in inclusive practices. While attitudes are generally favorable, the presence of barriers and only moderate institutional support suggests that teachers may lack the skills or confidence to implement inclusive strategies effectively. Courses on Universal Design for Learning (UDL), differentiated instruction, and behavior management should be embedded into teacher education programs ( Sharma et al., 2012). Furthermore, professional development should be ongoing and supported by mentoring and communities of practice within schools.

Another critical implication concerns parental and community involvement. The strong representation of parents in the study underscores the importance of family-school partnerships in achieving inclusive education. Policies and programs should encourage schools to engage parents in individual education planning (IEPs), decision-making, and awareness campaigns that reduce stigma and foster inclusive values ( Alnahdi et al., 2019; Slee, 2011). Lastly, the moderate score for perceived barriers suggests a need for infrastructure development, not just physical infrastructure such as ramps and sensory rooms, but also administrative and curriculum. Inclusion cannot succeed in an environment with rigid curricula, inflexible assessment systems, and minimal psychological support services. This is particularly true for districts like South and Central Jeddah, where population density may place additional pressure on already stretched resources.

### Limitations

Despite the strengths of this study, several limitations should be acknowledged. The geographic specificity of the sample, which focused solely on the city of Jeddah, limits the generalizability of the findings to other regions in Saudi Arabia. While Jeddah is a major urban center with diverse demographics, rural and remote areas may present entirely different challenges and opportunities in implementing inclusive education. Future studies should consider including participants from a broader cross-section of the Kingdom to enhance representativeness. Another limitation is the use of self-reported data, which may be subject to social desirability bias. Participants might have overreported their positive attitudes or underreported the presence of barriers due to cultural sensitivities or perceptions of institutional scrutiny. Although the anonymity of responses was maintained, qualitative follow-ups such as interviews or classroom observations would provide a more nuanced understanding of actual practices.

Moreover, while the study utilized robust statistical analyses, including regression and path analysis, it relied on observed variables and did not explore latent constructs. A more advanced SEM approach using latent variables might uncover deeper structural relationships and mediating effects between variables such as teacher efficacy, school leadership, and inclusive policy implementation. Future research should integrate such models and possibly include longitudinal designs to assess change over time. Lastly, the instrument used, although validated, was standardized and structured, possibly constraining more contextual or culturally nuanced responses. Future studies might include open-ended questions, or mixed method designs to capture the richness of experiences from diverse stakeholders, especially students with disabilities themselves, whose voices are often underrepresented.

## Conclusion

This study examined the landscape of inclusive education in Jeddah through the perspectives of teachers, special educators, principals, and parents. The aim was to quantify attitudes toward inclusion, identify perceived barriers, and evaluate the role of institutional support. The findings indicate that while attitudes toward inclusive education are generally positive, these attitudes exist within a system that still struggles with moderate levels of institutional support and persistent structural and pedagogical barriers. Descriptive statistics revealed that most respondents support the idea of inclusive education, with a mean attitude score of 3.85 out of 5. However, the presence of perceived barriers (M = 2.90) and only moderate institutional support (M = 3.23) highlights ongoing challenges. Inferential analyses, including ANOVA and t-tests, showed no significant differences in attitudes based on gender or professional role, suggesting a shared viewpoint across stakeholder groups.

Importantly, path analysis revealed significant predictive relationships: institutional support was found to influence attitudes, while barriers negatively impacted them positively. These findings underscore the importance of systemic support structures in fostering successful inclusion. Basic regression analysis failed to show significance, indicating that linear models may not fully capture the complexity of the relationships, further justifying the use of advanced techniques like path analysis. Although the study is geographically limited to Jeddah and based on self-reported data, it contributes valuable insights into how inclusive education is experienced at the school and community levels. The evidence suggests that while the philosophical foundation for inclusion is in place, practical and structural implementation remains uneven and in need of focused development.

### Recommendations

Based on the findings, several recommendations are proposed to strengthen inclusive education in Jeddah and similar urban areas of Saudi Arabia. First, institutional support systems should be reinforced through consistent funding, leadership commitment, and clear, inclusive education policies, along with adequate resources, assistive technologies, and trained staff. Second, teacher preparation and professional development must be enhanced by integrating inclusive pedagogy, Universal Design for Learning (UDL), and classroom differentiation into training programs, supported by ongoing mentoring. Improving infrastructure and accessibility is equally important; schools should be upgraded for physical and sensory access, especially in high-density districts such as South and Central Jeddah. Families and communities should also be actively engaged through partnerships and awareness campaigns to reduce stigma and promote acceptance. Moreover, national monitoring and accountability systems should be established to track inclusion indicators and ensure equitable service delivery. Expanding research into rural and remote areas will provide a more comprehensive understanding of the challenges associated with inclusive education across the country. These measures may help bridge the gap between policy and practice, fostering a more inclusive and equitable education system aligned with Saudi Arabia’s Vision 2030.

## Data Availability

Zenodo: *Navigating Barriers and Building Pathways: Inclusive Education for Children with Special Needs.* DOI: https://doi.org/10.5281/zenodo.17429112 ( Aldhilan et al., 2025). This project contains the following underlying data:
• Data Analysis.docx: Complete output of descriptive and inferential statistics (e.g., t-test, ANOVA, SEM) Zenodo: *Navigating Barriers and Building Pathways: Inclusive Education for Children with Special Needs.* DOI: https://doi.org/10.5281/zenodo.17429112 ( Aldhilan et al., 2025). This project contains the following extended data:
• Questionnaire.docx: Structured Likert-scale-based tool used in the study • Consent Form.docx: Template form used to obtain informed consent •
Extended_Data_Descriptions.docx: Description of files and use Data are available under the terms of the Creative Commons Attribution 4.0 International license (CC-BY 4.0).
