# Supplementary material for: Navigating Barriers and Building Pathways: Inclusive Education for Children with Special Needs
Source: F1000Res. 2025 Nov 25;14:1203. Originally published 2025 Nov 4. [Version 2] doi: 10.12688/f1000research.172211.2 (PMC12595298; doi:10.12688/f1000research.172211.2)
Supplement: Peer review history

# Reviewer report: Reviewer response for version 2

**DOI:** 10.5256/f1000research.191698.r435830

**Published:** 2025-11-27

**Authors:** Farahat Ali

**Affiliations:** 1 Beaconhouse National University, Lahore, Pakistan

No comment. Everything seems fine.

Is the work clearly and accurately presented and does it cite the current literature?

Yes

If applicable, is the statistical analysis and its interpretation appropriate?

Yes

Are all the source data underlying the results available to ensure full reproducibility?

Yes

Is the study design appropriate and is the work technically sound?

Yes

Are the conclusions drawn adequately supported by the results?

Yes

Are sufficient details of methods and analysis provided to allow replication by others?

Yes

Reviewer Expertise:

Media and Education

I confirm that I have read this submission and believe that I have an appropriate level of expertise to confirm that it is of an acceptable scientific standard.

# Reviewer report: Reviewer response for version 1

**DOI:** 10.5256/f1000research.189926.r430271

**Published:** 2025-11-10

**Authors:** Farahat Ali

**Affiliations:** 1 Beaconhouse National University, Lahore, Pakistan

The article, "Navigating Barriers and Building Pathways: Inclusive Education for Children with Special Needs," offers a comprehensive quantitative analysis of inclusive education in Jeddah, Saudi Arabia. The research is well-motivated, addressing persisting challenges in the Saudi context within the framework of Vision 2030 and global best practices. The study design is robust, with clearly stated objectives, thorough literature grounding, and the use of well-established theoretical models (Social Model of Disability and Bronfenbrenner’s Ecological Systems Theory).

- The manuscript is clearly written, well-structured, and thoroughly cites current and relevant literature.
- The sampling and survey methodology are rigorous and appropriate, with good attention to representativeness and validity in the Jeddah context.
- Methodological details, including statistical analyses (descriptive stats, ANOVA, t-tests, regression, and path analysis), are meticulously presented, allowing for clear understanding and potential replication.
- The conclusions are well-supported by the data and analyses, and limitations are transparently acknowledged.
- The discussion not only contextualizes results within international and regional scholarship but also offers practical, evidence-based recommendations.
- The availability of all underlying data, extended data, and research instruments ensures the highest standards of transparency and reproducibility.
- The paper’s emphasis on institutional support and systemic reform as keys to successful inclusive education is timely and aligns with international calls for systemic change.
- The policy recommendations are actionable and grounded in the findings, which will be valuable for both policymakers and practitioners.
- The study’s clear alignment with Saudi Arabia’s Vision 2030 and its acknowledgement of localized challenges add real-world relevance.
- The inclusion of diverse stakeholder perspectives (teachers, administrators, parents) strengthens the paper’s contribution.

Overall, this is an excellent and timely contribution to the field of inclusive education research in Saudi Arabia and the Middle East. The study is scientifically sound, methodologically robust, and practically relevant. I strongly endorse its indexing and commend the authors for their valuable work.

Is the work clearly and accurately presented and does it cite the current literature?

Yes

If applicable, is the statistical analysis and its interpretation appropriate?

Yes

Are all the source data underlying the results available to ensure full reproducibility?

Yes

Is the study design appropriate and is the work technically sound?

Yes

Are the conclusions drawn adequately supported by the results?

Yes

Are sufficient details of methods and analysis provided to allow replication by others?

Yes

Reviewer Expertise:

Media and Education

I confirm that I have read this submission and believe that I have an appropriate level of expertise to confirm that it is of an acceptable scientific standard.

# Reviewer report: Reviewer response for version 1

**DOI:** 10.5256/f1000research.189926.r430272

**Published:** 2025-11-07

**Authors:** Megren Abdullah Altassan

**Affiliations:** 1 University of Business and Technology, Jeddah, Saudi Arabia

The presents a comprehensive and thoughtfully designed quantitative investigation into the state of inclusive education for children with special needs in Jeddah, Saudi Arabia. Drawing data from 550 participants, including teachers, special educators, school leaders, and parents, the study explores perceptions of inclusion, levels of institutional support, and the challenges that hinder effective practice within educational environments.

 Anchored in the Social Model of Disability and Bronfenbrenner’s Ecological Systems Theory, the research offers a multidimensional lens through which the social, cultural, and institutional factors shaping inclusive education are examined. The authors employ a stratified random sampling technique and apply a range of advanced statistical analyses such as ANOVA, regression, and path analysis, ensuring methodological rigor and analytical precision.

 Findings reveal generally positive attitudes toward inclusion yet highlight persistent barriers and insufficient institutional support as key impediments to implementation. Notably, the path analysis demonstrates that institutional support significantly enhances positive attitudes, while perceived barriers exert a negative effect, offering strong empirical evidence to guide policy and practice.

 The manuscript stands out for its clarity, coherence, and academic sophistication, and its alignment with Saudi Arabia’s Vision 2030 underscores its policy relevance and national importance. Ethical standards are meticulously observed, and the open availability of data via Zenodo strengthens the study’s transparency and reproducibility.

 Overall, this article constitutes a substantive and original contribution to the field of inclusive education, combining theoretical depth with practical significance. It represents a methodologically sound and well-articulated study that merits recognition and acceptance in its present form.

Is the work clearly and accurately presented and does it cite the current literature?

Yes

If applicable, is the statistical analysis and its interpretation appropriate?

Yes

Are all the source data underlying the results available to ensure full reproducibility?

Yes

Is the study design appropriate and is the work technically sound?

Yes

Are the conclusions drawn adequately supported by the results?

Yes

Are sufficient details of methods and analysis provided to allow replication by others?

Yes

Reviewer Expertise:

Special Needs Education, Inclusive Education, Special Education Management

I confirm that I have read this submission and believe that I have an appropriate level of expertise to confirm that it is of an acceptable scientific standard.

# Reviewer report: Reviewer response for version 1

**DOI:** 10.5256/f1000research.189926.r430268

**Published:** 2025-11-07

**Authors:** Madiha Khadim

**Affiliations:** 1 GC Women University Faisalabad, Punjab, Pakistan

The article presents an insightful and well-structured quantitative study exploring the current landscape of inclusive education for children with special needs in Jeddah, Saudi Arabia. Drawing on responses from 550 participants including teachers, special educators, administrators, and parents, the study examines perceptions of inclusion, institutional support, and existing barriers within educational settings.

 The research is grounded in the Social Model of Disability and Bronfenbrenner’s Ecological Systems Theory, which together provide a rich conceptual framework for understanding the social and institutional dynamics influencing inclusive education. The authors have adopted a rigorous methodological approach using stratified random sampling and robust statistical analyses such as ANOVA, regression, and path analysis, which lend credibility and precision to the findings. Results indicate that while overall attitudes toward inclusive education are positive, moderate levels of barriers and limited institutional support continue to challenge effective implementation.

 Importantly, the path analysis reveals that institutional support strongly enhances positive attitudes, whereas perceived barriers have a negative influence, providing valuable evidence for policymakers and practitioners. The article demonstrates exceptional clarity, coherence, and scholarly depth, and its alignment with Saudi Arabia’s Vision 2030 further highlights its national significance. Ethical considerations are properly addressed, and the availability of all underlying data through Zenodo ensures transparency and full reproducibility. Overall, this study represents a high-quality contribution to the field of inclusive education and offers both theoretical and practical insights for educators, administrators, and policymakers. It is a well-executed, well-written, and scientifically sound piece of research that deserves appreciation and acceptance in its current form.

Is the work clearly and accurately presented and does it cite the current literature?

Yes

If applicable, is the statistical analysis and its interpretation appropriate?

Yes

Are all the source data underlying the results available to ensure full reproducibility?

Yes

Is the study design appropriate and is the work technically sound?

Yes

Are the conclusions drawn adequately supported by the results?

Yes

Are sufficient details of methods and analysis provided to allow replication by others?

Yes

Reviewer Expertise:

Inclusive Education, Special Needs education, Educational Leadership and Management, Sustainable development,

I confirm that I have read this submission and believe that I have an appropriate level of expertise to confirm that it is of an acceptable scientific standard.
